# Variation in Weed Seed Fate Fed to Different Holstein Cattle Groups

**DOI:** 10.1371/journal.pone.0154057

**Published:** 2016-04-22

**Authors:** Salman Rahimi, Hamid Rahimian Mashhadi, Mehdi Dehghan Banadaky, Mohsen Beheshtian Mesgaran

**Affiliations:** 1 Department of Agronomy and Plant Breeding, College of Agriculture Science and Engineering, University of Tehran, Karaj, Iran; 2 Department of Animal Science, College of Agriculture Science and Engineering, University of Tehran, Karaj, Iran; 3 School of BioSciences, The University of Melbourne, Victoria, Australia; Instituto de Agricultura Sostenible (CSIC), SPAIN

## Abstract

Weed seeds may maintain their viability when passing through the digestive tract of cattle and can be therefore dispersed by animal movement or the application of manure. Whether different cattle types of the same species can cause differential weed seed fate is largely unknown to us particularly under non-grazed systems similar to Holstein-Friesian dairy farming. We investigated the effect on the seed survival of four weed species in the digestive tracts of four groups of Holstein cattle: lactating cows, feedlot male calves, dry cows and growing heifers. The weed species used were *Cuscuta campestris*, *Polygonum aviculare*, *Rumex crispus* and *Sorghum halepense*. Cattle excretion was sampled for recovery and viability of seeds at four 24 hourly intervals after seed intake. The highest seed recovery occurred two days after seed intake in all cattle groups. Averaged over weed species, dry and lactating cows had the lowest and highest seed recovery of 36.4% and 74.4% respectively. No significant differences were observed in seed recovery of the four weed species when their seeds were fed to dry cows. Based on a power model fitted to seed viability data, the estimated time to 50% viability loss after seed intake, over all cattle groups ranged from 65 h (*R*. *crispus*) to 76 h (*P*. *aviculare*). Recovered seeds from the dung of feedlot male calves showed the highest mortality among cattle groups. Significant correlation was found between seed viability and ruminal pH (r = 0.86; *P<0*.*05*). This study shows that management programs aiming to minimize weed infestation caused by livestock should account for the variation amongst cattle groups in seed persistence. Our findings can be used as a guideline for evaluating the potential risk of the spread of weeds via the application of cattle manure.

## Introduction

The seeds of many weed species can remain viable after passing through the digestive tract of livestock [1, 2]. The dungs and manures of different species of livestock have been found to contain a variable number of viable weed seeds [3, 4], which makes livestock a major agent of weed seeds dispersal in both grazing [5, 6] and non-grazing systems [7, 8].

Dairy manure, commonly applied in croplands either directly or as a compost can be contaminated with weed seeds [9, 10] and thus can result in further escalation of weeds in farms. With the current increase in the adaptation of organic production systems [11], weed infestations through livestock are expected to become greater than in the past, as these systems are largely reliant on the use of organic amendments such as manure [12]. Indeed, weeds are known to be a major constraint to the productivity of organic farms [13].

Infestations caused by manure applications can be highly variable because seed recovery and viability after digestion vary considerably depending on the livestock type [14], feed [15] and plant species [16]. For example, physical damage to seeds depends on the degree of mastication, which varies among livestock species. Sheep and goats exert more damage to the seed than cattle do, and [17] feed properties including forage/concentrate ratio, particle size, quantity and digestibility [2, 15] can affect seed viability and recovery though changes in rumen microbial population, ruminal pH and the passage rate of rumen fluid [18–20]. For example, seed recovery for highly digestible feed was higher than less digestible feed due to a marked reduction in the retention time in the rumen/digestion tract [15].

Seed properties such as the hardness of seed coat, seed size, shape and specific gravity [16, 17, 21] are important to the survival of seeds passing through the digestive tract of livestock. Small-round seeds with smooth exteriors [6, 22], seeds with high specific gravity and impermeable seeds [16] typically have high recovery and survival.

Previous studies have focused on grazing livestock and pasture plant species, however, only a few studies have examined the fate of weed seeds under non-grazed systems. Previous studies have shown that the dispersibility of seeds varies among different animals [23–25] yet whether different cattle types within the same species can cause differential weed seed fate is poorly understood. The most popular milk-producing dairy cattle breed globally is the Holstein-Friesian [26], which are classified into four major groups: 1- lactating cows, 2- feedlot male calves, 3- dry cows, and 4- growing heifers. As these cattle groups vary in physiological properties of their digestive tract (particularly in the reticulum-rumen function) [20] and receive different daily diets, we hypothesized that the fate of weed seeds, measured in terms of recovery and viability, will depend on the cattle type. We tested this hypothesis using four weed species: *Cuscuta campestris* Yuncker., *Polygonum aviculare* L., *Sorghum halepense* (L.) Pers, and *Rumex crispus* L. These weed species are common in crops from where the cattle feeds are sourced and they also vary in seed (physical) properties. The use of weeds with contrasting seed properties (Table 1) could allow us to examine the association between seed traits and the propensity to survive the digestive tract.

## Materials and Methods

### Seed Source

The seeds of four weed species were collected at maturity from infested fields in Karaj, Iran (latitude 35˚ 48' N; longitude 50˚ 57' E) in early September 2009 (Table 1). The selected species are amongst the most abundant weed species in the crops which are utilized to produce the livestock feed in many regions of Iran [27]. The seeds were cleaned by hand and stored indoor for two weeks until used in the experiment. The species vary in seed dimension, weight and specific gravity as shown in Table 1.

**Table 1:** Seed properties of four weed species used to examine recovery and survival after ingestion by Holstein cattle.

Species	Seed lenght×width (mm)	1000-seed weight (g)	Specific gravity	Initial seed viability (%)
*Cuscuta campestris*	2×2	1.3	1.25	91.5
*Polygonum aviculare*	2.5×2	1.2	1.05	91.7
*Rumex crispus* *	4×3	1.5	0.34	100
*Sorghum halepense* *	4.5×3	4	1.15	92.2

* Size was measured for fruit.

Prior to seed feeding study, to test initial seed viability, four replicates of 25 seeds from each weed species were placed on a Whatman No. 2 filter paper moistened with 5 mL of distilled water, in an 8.0 cm diameter Petri dish. Dishes were incubated for 14 d at temperatures and photoperiod conditions optimal for the germination of individual weed species: these were 30°C with an 8 h photoperiod for *C*. *campestris* [28], 20 /10°C (light/dark) with a 8 h photoperiod for *P*. *aviculare* [29], 25°C in continuous darkness for *R*. *crispus* [30], and 30°C in continuous darkness for *S*. *halepense* [31]. At the end of the germination assay, the viability of non-germinated seeds was examined using a tetrazolium chloride (TZ) test [32], whereby the seed coat was scarified with a scalpel to expose the embryo to 2.0% 2,3,5-triphenyl tetrazolium chloride solution (Sigma-Aldrich, St. Louis, USA), pH 7.0, for 48 h at 20°C. Seeds stained red were regarded alive and were counted. The number of viable seeds was calculated as the sum of seeds that had germinated and non-germinated seeds with TZ solution-stained embryos.

### Seed Feeding and Sampling

The seed-feeding study was performed in the Animal Science Research Station of the University of Tehran in October 2009, to quantify seed recovery and seed viability in four different Holstein cattle groups: 1- lactating cows (weight 600±50 kg, 24–28 months old, days in milk 50 d), 2- feedlot male calves (weight 410±30 kg, 10–12 months old), 3- dry cows (weight 650±50 kg, 35–38 months old), and 4- growing heifers (weight 400±25 kg; 12–15 months old). Department of Animal Science in University of Tehran is responsible for all studies performed in the Research Station. The seed sampling did not involve endangered or protected species. Animals were cared for in accordance with the guidelines of the Iranian Council on Animal Care [33]. No permit was required from the above authority as our study involved no treatment on animal daily cares. All sampling procedures were performed without any stress to the animals.

Four individuals (indicative of four replicates) from each of the groups were housed in individual tie stalls for 15 d. The cattle were acclimatized in their stalls for 10 days before five days of seed-feeding began. All cattle groups were fed according to the recommendations of the Nutrient Requirements of Dairy Cattle [34] as shown in Table 2. The lactating cows and feedlot male calves were fed twice daily at 0700 and 1530 ad libitum, but feeding times were restricted for dry cows and growing heifers. All cattle groups received continual access to water.

**Table 2:** Ingredients and chemical composition of the diet of four groups of Holstein cattle.

	Lactating cow	Feedlot male calf	Dry cow	Growing heifer
Feed intake dry matter (kg d^-1^)	20	12	10	10
Ingredient, % of dry matter				
Lucerne hay	21.08	12.11	12.76	34.32
Maize Silage	15.69	21.31	33.67	42.79
Wheat straw	0	0	34.01	0
Beet pulp	9.73	0	0	0
Oilseed rap meal	3.75	3.20	4.57	5.34
Soya bean meal	12.84	0	2.28	2.67
Wheat grain	3.75	0	0	0
Maize grain	7.49	2.26	0	0
Barley grain	16.94	44.61	4.89	5.72
Wheat bran	0	5.33	2.93	3.43
Rice bran	0	7.98	3.91	4.58
Cotton seed	2.68	0	0	0
Maize gluten	0.54	0	0	0
Fat powder*	1.61	0.47	0	0
Vitamin-mineral mix	3.69	1.73	0.87	1.02
Trace mineralized salt	0.21	1.00	0.11	0.13
**Chemical composition**^**†**^				
CP, % of dry matter	17.55	14	13.3	14.4
NDF, % of dry matter	34.9	41.2	49	45
ADF, % of dry matter	20.48	18	29	25
NFC, % of dry matter	34.5	38	25	30
Ash, % of dry matter	9.94	10	8.69	9
NE_L_, Mcal/kg of dry matter	1.68	-	-	-
ME_M_, Mcal/kg	-	1.7	-	-

* As prilled protected fat; Energizer-10, (IFFCO, Johor, Malaysia).

† Calculated based on the data provided by National Research Council (2001).

CP: Crude Protein; NDF: Neutral Detergent Fiber; ADF: Acid Detergent Fiber; NFC: Non-fiber Carbohydrate; NE_L_: Net Energy for milking; ME_M_: Net Energy for maintenance.

On the 11^th^ day, the seeds of four weed species were mixed with 0.5 kg aromatic calf concentrate and sugar beet molasses, and then fed as a supplement to each animal. For each weed species, 1500 seeds per kg of feed was added to the cattle diets. Based on the amount of feed given to each group (Table 2), the total number of seeds fed (summed over the four weed species) were 120000, 60000, 60000, and 72000 seeds for lactating cows, dry cows, growing heifers, and feedlot male calves, respectively.

Total dung output for each animal was collected and weighed every 24 h for four consecutive days. One kg of the daily homogenized excretion was randomly sampled for seed recovery and viability testing. An additional sample (1 kg) of daily dung output was oven dried at 68˚C for 48 h and weighed to determine the dry matter content. The pH of ruminal fluid was measured on the 14^th^ d of trials at 0700 am before the morning meal, by taking 50 mL of rumen fluid from the ventral sac using a vacuum pump. Ruminal pH was measured immediately after sampling using a portable pH meter (Metrohm, Herisau, Switzerland).

### Seed Recovery and Viability

Daily samples of the dung output were immediately washed through a 60 mesh sieve under tap water. We used a mesh size of 0.25 mm, which is smaller than the smallest seed in this experiment, to ensure zero seed loss. The residuals (left in the sieve) were dried indoors on a thick layer of paper and then run through an air blower to remove finer materials. Undamaged weed seeds were separated and counted using a 10X magnifying glass on a marble slate. For each weed species and cattle group the daily seed recovery rate (*SR*) was calculated as follows:

$$SR\left(\%\right)=\frac{N_{sample}\times DDM}{N_{add}}\times 100$$ (1)

where *N*_*sample*_ is the number of undamaged seeds extracted from the daily 1 kg sample, *DDM* indicates the total daily dry matter of dung output and *N*_*add*_ is the total number of seeds added to the feed at the beginning of the experiment.

The viability of extracted (undamaged) seeds was tested in the same way as with the fresh seeds. The percent seed viability was calculated as the total number of viable seeds passed through the digestive tract, divided by the total number of viable seeds fed to cattle at the beginning of experiment (see Table 1 for the initial seed viability of each species). Based on the percentage of seed recovery and viability, we also calculated total recovered viable seed (RVS) for each weed species and cattle group in four consecutive days (*i* = 1, 2,3, 4) as follows:

$$RVS\left(\%\right)=\sum_{i=1}^{4}\frac{NR_{i}\times SV_{i}}{NV_{add}}\times 100$$ (2)

where *NR*_*i*_ is the number of undamaged seeds extracted from the 1 kg sample for day *i*, *SV*_*i*_ indicates the viable seed fraction for day *i* and *NV*_*add*_ is the total number of viable seeds added to the feed at the beginning of the experiment.

### Experimental Design and Statistical Analysis

The experiment was conducted as a factorial, encompassing the full cross of cattle type (four groups) by weed species (four species), within a randomized complete block design with four replications. Each animal was regarded as a block whereby measurements of seed recovery and viability were taken over four consecutive days. There was no need for data transformation as residuals were normally distributed and homogenous. A repeated measurement analysis of data was performed using PROC MIXED procedure of SAS (Version 9.2; SAS Institute, Cary, NC, USA) to test the significance of the effects of the cattle groups, weed species, time and their interactions on seed recovery and seed viability. The REPEAT statement of SAS was used to test for the effect of time using an unstructured covariance matrix with block as a random effect. Significant differences among means were identified by Least Significant Difference (LSD) at the 0.05 level.

The cumulative seed recovery (% of seed fed) over time, *CR*, was described by a three parametric sigmoidal model:

$$CR\left(\%\right)=\frac{a}{1+\text{exp}-\left(\frac{t-t_{R50}}{b}\right)}$$ (3)

where *a* is the maximum *CR* that occurs on the last sampling day (i.e. four days after seed intake), *t* is time, *t*_*R*50_ is the time to reach 50% of *a* and *b* indicates the steepness of the curve. Changes in percent seed viability over time were best described by a power model:

$$V\left(\%\right)=\{\begin{array}{l} \beta \;t^{\alpha }+c\;if\;t>{\left(-c/a\right)}^{\frac{1}{b}}\\ 0\;else \end{array}$$ (4)

where *c* is the maximum viability occurring at *t* = 0 and was fixed at 100%, while *β* indicates the rate (steepness) of viability loss over time and *α* is a shape parameter determining the degree of curvature (e.g. *α* = 1 model reduced to a linear regression model). Note that at *t* ≥ (−*c*/*β*)^1/*α*^ the predicted viability will be zero, which in conjugation with a constant *c* = 100, the model does not predict unrealistic negative or > 100% viability percentages. From Eq. 4, we have derived the half-life of seeds fed to different cattle groups by using:

$$t_{V50}=50^{1/\alpha }{\left(\frac{-1}{\beta }\right)}^{1/\alpha }$$ (5)

where *t*_*V*50_ measures the length of time until 50% loss in seed viability. The above models were fitted to the data using PROC NLIN of SAS. Additionally, the correlation between seed viability and ruminal pH was calculated using PROC CORR of SAS (Version 9.2; SAS Institute, Cary, USA).

## Results

### Seed Recovery

**Fig 1:**
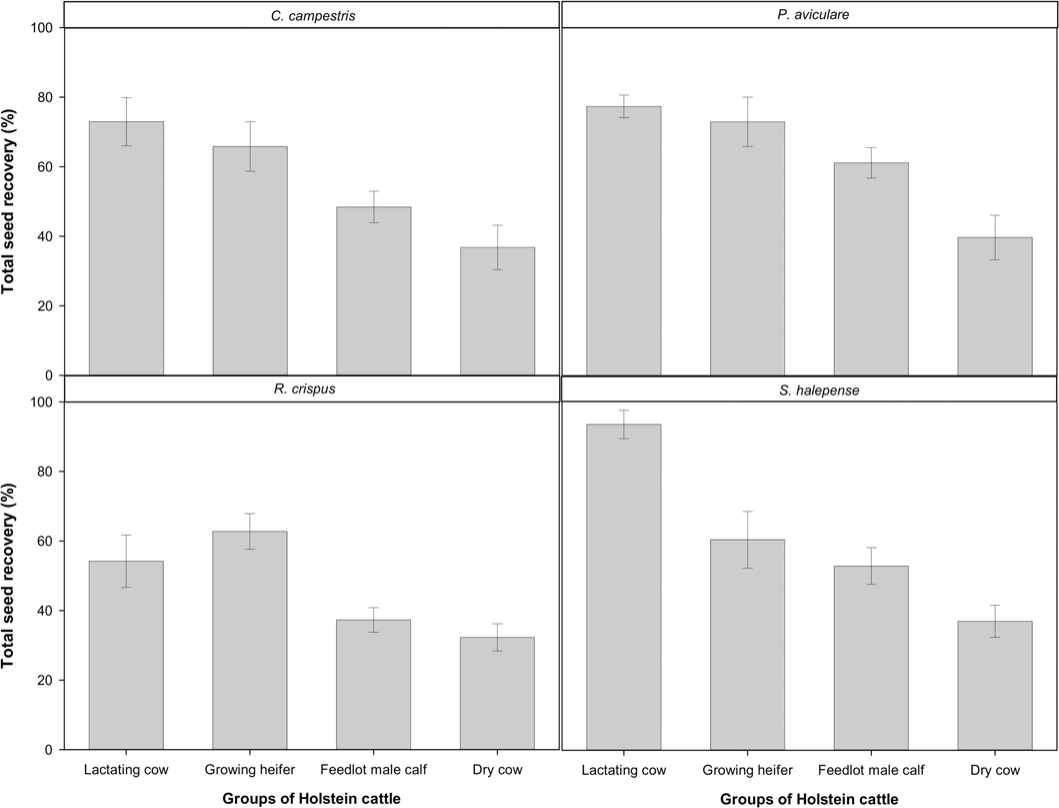
Total seed recovery (after four days) of four weed seeds fed to different groups of Holstein cattle (vertical bars denote one standard error).

All main effects and their interactions were significant for seed recovery (Table 3). Total seed recovery (summed over the four sampling dates) for all weed species was lowest in dry cows and never exceeded 45%. The highest percent recovery was observed with lactating cows with the only exception of *R*. *crispus* (Fig 1). For this species, seed recovery was consistently low regardless of the cattle type as opposed to *S*. *halepense*, which exhibited a high percent recovery particularly when fed to lactating cows (94% seed recovery). Analysis of within Holstein cattle group showed that seed recovery dose not vary among weed species in the dry cows and growing heifers (Table 3). No significant difference was observed, averaged over cattle types, between the total seed recovery of *P*. *aviculare* and *S*. *halepense*.

**Table 3:** ANOVA results for the effects of cattle group (G), weed species (W), time (T) and their interactions on the seed recovery, viability and recovered viable seed. Also shown is the within cattle group analysis of weed species differences for describing the G by W interaction.

		Seed recovery		Seed viability		Recovered viable seed		
Source of Variation	d.f.	F value	*Pr > F*	F value	*Pr > F*	F value		*Pr > F*
Group of HC(G)	3	65.92	**< .0001**	188.06	**< .0001**	96.38		**< .0001**
Weed species (W)	3	12.27	**< .0001**	58.25	**< .0001**	23.93		**< .0001**
G×W	9	3.70	**0.0003**	2.19	**0.0244**	2.85		**0.0035**
Time (T)	3	781.75	**< .0001**	6555.58	**< .0001**	870.39		**< .0001**
G×T	9	127.75	**< .0001**	29.92	**< .0001**	118.23		**< .0001**
W×T	9	14.89	**< .0001**	25.78	**< .0001**	10.06		**< .0001**
G×W×T	27	5.29	**< .0001**	2.37	**0.0004**	5.34		**< .0001**
**G×W interaction sliced by groups of Holstein cow**								
Lactating cow	3	15.33	**< .0001**	22.33	**< .0001**		17.96	**< .0001**
Feedlot male calf	3	5.77	**0.0009**	4.91	**0.0026**		8.32	**< .0001**
Dry cow	3	0.55	0.6456	25.90	**< .0001**		1.50	0.2167
Growing heifer	3	1.71	0.1657	11.91	**< .0001**		4.72	**0.0033**

Bold numbers indicate significant effects.

**Fig 2:**
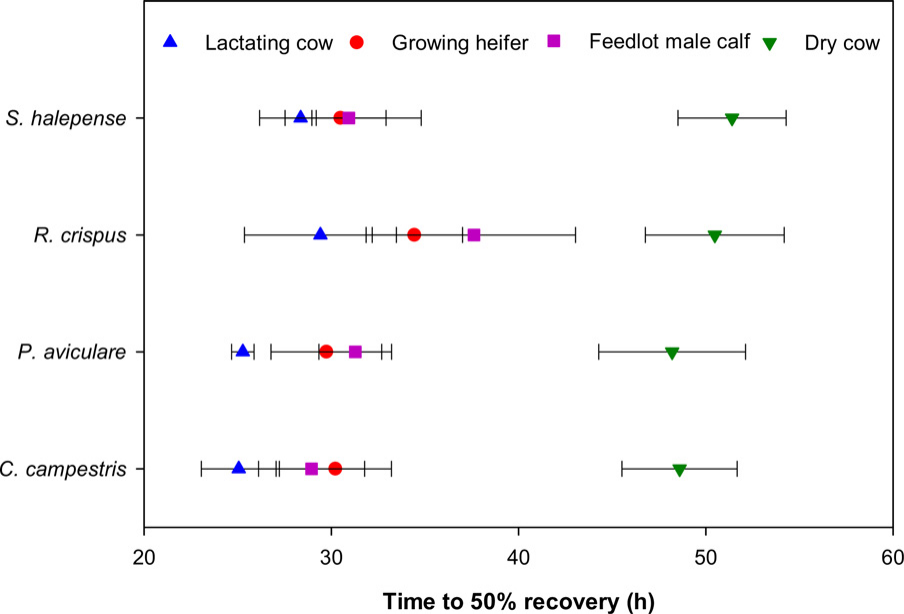
Time to 50% seed recovery (*t*_*R*50_; Eq 3) for four weed seeds passed through the digestive tract of the four Holstein cattle groups (horizontal bars denote one standard error).

The recovery time (i.e. amount of time required to recover 50% of the total seeds recovered by the end of sampling), as inferred from the parameter *t*_*R*50_ (Eq 3), varied from 25 h in lactating cows for *C*. *campestris*, to 51 h in dry cows for *S*. *halepense* (Fig 2). Recovery time varied across cattle types in a similar manner to that of the percent seed recovery, whereby dry cows had the slowest passage rate, while lactating cows had the fastest passage rate. That is, the recovery time for dry cows was approximately twice as long as that of lactating cows. Other cattle types, growing heifers and feedlot male calves, were intermediate in this respect. Recovery times tended to be longer for *R*. *crispus* compared to other weed species, particularly when fed to growing heifers (Fig 2).

### Seed Viability

**Fig 3:**
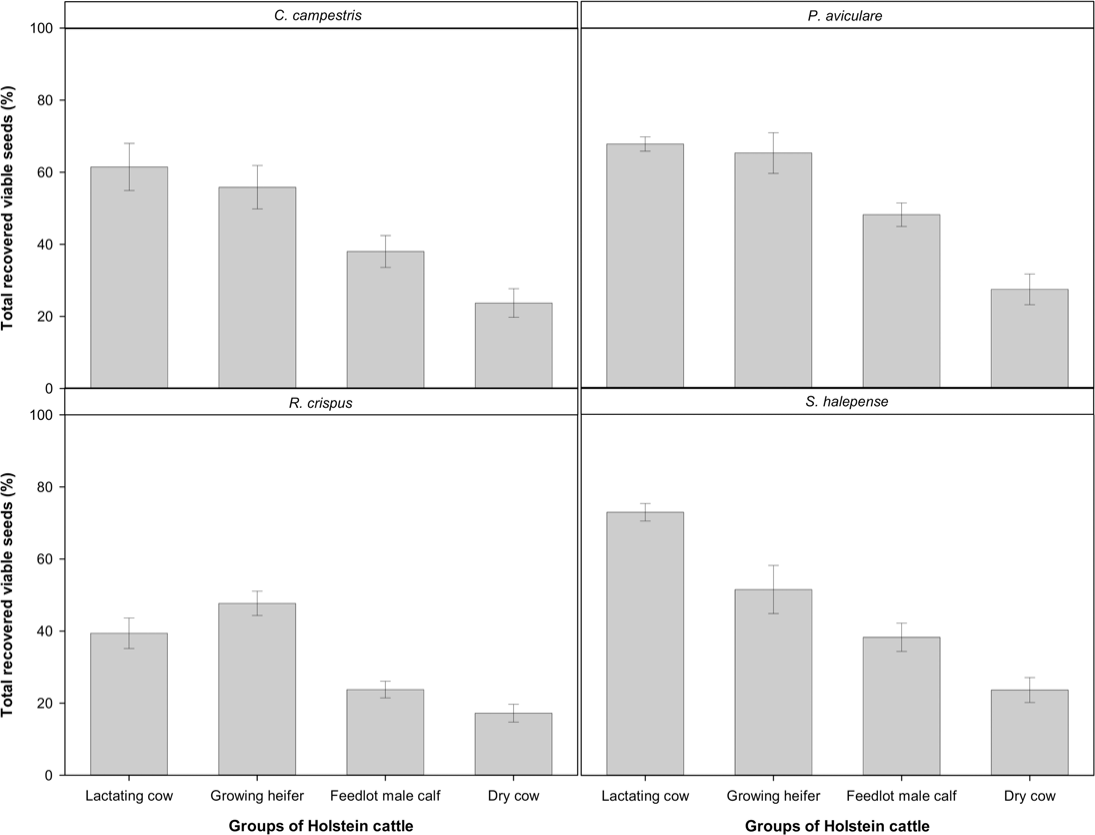
Total recovered viable seeds (summed over four consecutive days) of the four weed seeds passed through the digestive tract of different groups of Holstein cattle (vertical bars denote one standard error).

All main effects and their interactions were significant for the seed viability and recovered viable seed measured over the four consecutive dates (Table 3). The highest value of total recovered viable seed (73%) was observed with *S*. *halepense* when fed to lactating cows but was not significantly different from *P*. *aviculare* in this group and growing heifers. It was minimal (17%) in *R*. *crispus* when fed to dry cows (Fig 3). For all weed species (except *R*. *crispus*), recovered viable seeds showed a consistent decreasing trend with feedlot lactating cows > growing heifers > feedlot male calves > dry cows. Averaged over the four cattle groups, the most persistent seeds were *P*. *aviculare* with 52% viability of fed seeds, the least persistent fed seeds were those of *R*. *crispus* with 32% viability (Fig 3).

**Fig 4:**
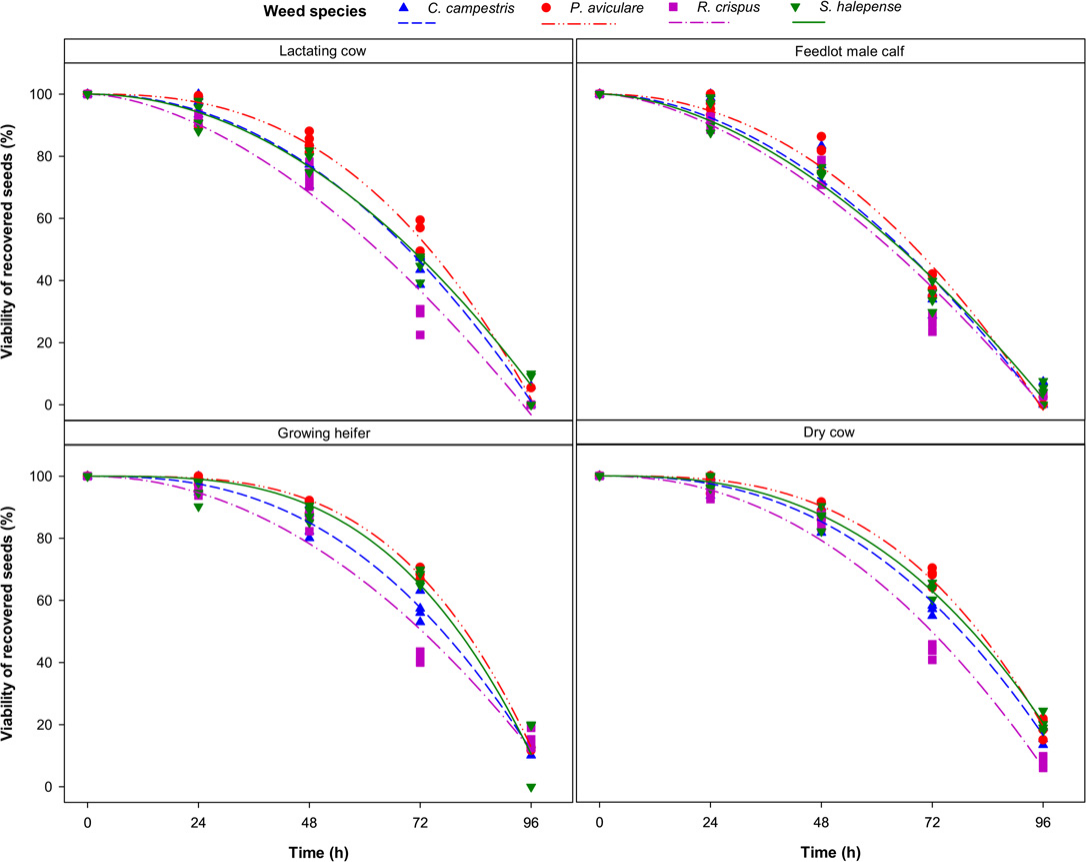
Changes in viability of the four weed seeds over time (as h after seed intake) for four groups of Holstein cattle. Symbols are observed data and lines are fitted values obtained from Eq 4.

The power model (Eq 4) provided adequate fits to viability data over time (S1 Table; Fig 4). Seed viability declined with time after seed intake, however, the rate and magnitude of reductions varied across weed species and cattle groups (Fig 4). There were few changes in seed viability during the initial times for growing heifers and dry cows, whilst viability loss was more rapid in other cattle groups (Fig 4).

**Fig 5:**
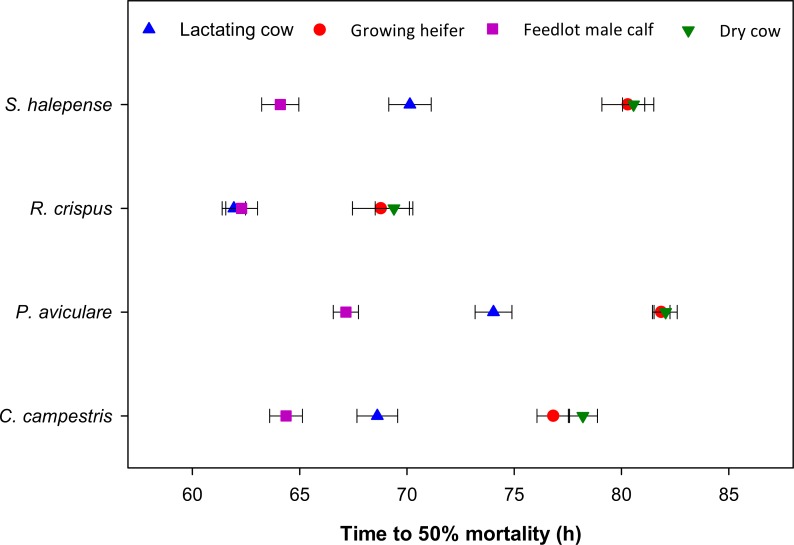
Time (as h after seed intake) to 50% mortality (half-life; *t*_*V*50_, Eq 5) for four weed seeds passed through the digestive tract of four Holstein cattle groups (horizontal bars denote one standard error).

We also estimated the half-life of seeds (*t*_*V*50_) fed to different cattle groups using Eq 5. The half-time, *t*_*V*50_, varied from 62 h for *R*. *crispus* in lactating cows and feedlot male calves, to 82 h for *P*. *aviculare* in dry cows (Fig 5). All weed species had a longer half-life when fed to dry cows and growing heifers, as shown by larger *t*_*V*50_ values (Fig 5). Seed mortality was fastest in feedlot male calves, where seeds had a half-life of 64 h. Although this parameter indicates higher seed mortality in feedlot male calves than growing heifers, but the total recovered viable seed was greater in growing heifers (Fig 1).

**Fig 6:**
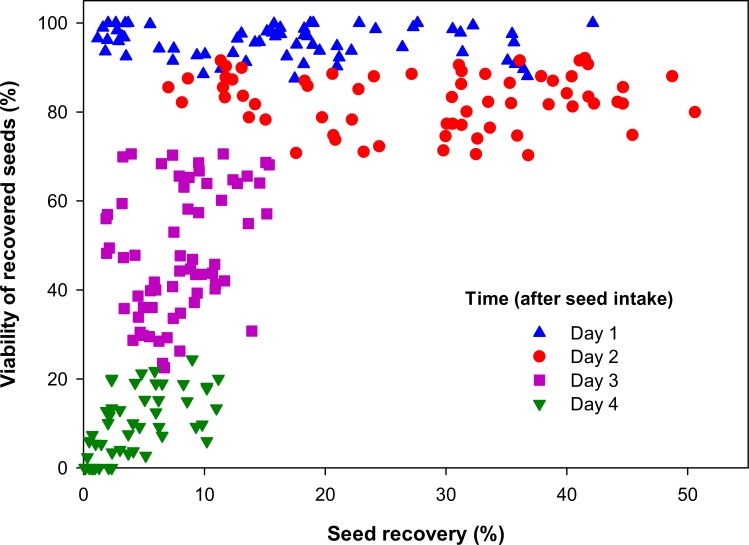
Relationship between seed recovery and seed viability for data collected over four consecutive days.

The relationship between seed recovery and viability was curvilinear (Fig 6) and showed a predictable pattern over time. Recovery was highly variable for the first two sampling days, ranging from 2% to 50%, and these seeds had viabilities as high as 70% to 100%. Conversely, for sampling 3 and 4 days after intake, the daily seed recovery was less variable but did not exceed 20%, while seed viability was highly variable and ranged from 0% to 70%. By four days after seed intake very few seeds were recovered (< 12%) and the majority were dead (< 24% viability).

**Fig 7:**
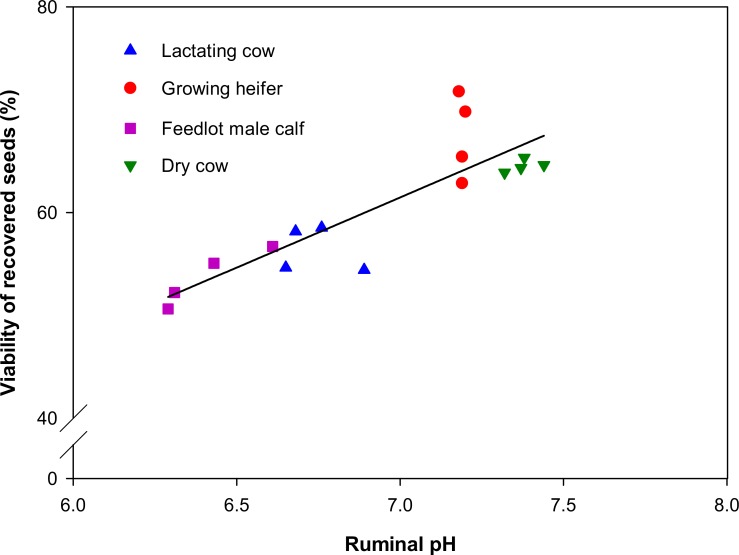
Relationship between seed viability and ruminal pH of four Holstein cattle groups.

A significant positive correlation was found between seed viability and ruminal pH with r = 0.86 (*P*<0.05; Fig 7). The pH was higher in dry cows and growing heifers than in feedlot male calves and lactating cows, and was also associated with higher seed viability.

## Discussion

This study showed that seed recovery and viability, as well as passage time through the digestive tract, can differ markedly between cattle types of the same livestock species. However, not all weed species showed a similar response.

The passage time and ruminal retention time of feed (and seeds within it) are determined principally by the frequency and amount of feed consumed, forage physical form, concentrate/forage ratio and forage fiber content [35]. Bodmer & Ward (2006) found a positive linear relationship between seed survival and animal body size [36], however, our higher seed recovery observed with growing heifers and feedlot male calves with small body sizes, rather than with dry cows with large body sizes. It seems that the amount of feed intake is more important than the body size. The amount of feed intake for lactating cows was approximately twice as much as that of the other cattle groups (Table 2). Increasing the amount of feed reduced the retention time while accelerating the flow through the reticulo-rumen, which in turn resulted in high seed output rate (as measured by time to 50% recovery) as observed in lactating cows (Fig 2).

Furthermore, diets with a high digestibility, i.e. with higher concentrate/forage ratios and lower levels of neutral detergent fiber (NDF), and acid detergent fiber (ADF) pass more quickly through the digestive tract of ruminants [20, 37]. For example, in lactating cows, such higher digestibility can result in higher seed recoveries than in other cattle groups (Fig 1). In our study, although concentrate/forage ratio was much lower in growing heifers, seed recovery in this group was higher than in feedlot male calves (Fig 1). Only 10% of ingested seed was recovered with low-digestibility diets compared to 28% with high-digestibility diets in sheep [15]. Wheat straw, which constituted 34% of the diet in dry cows (Table 2), can encourage chewing and increase ruminal retention time due to its high fiber content [18].

The recovery of seeds also varied among weed species, which can be attributed to their differences in physical characteristics (Fig 2). Gardener *et al*. (1993a) found a strong positive correlation between the specific gravity of seed and the rate of passage through the digestive tract of cattle, however, seed size was only weakly positively correlated with the passage time [16]. We found lower seed recovery in *R*. *crispus* than *S*. *halepense* despite the two species having seeds of the same size (Table 1; Fig 2). However, this difference in recovery can be explained by the differences in specific gravity between the two seed types, in that *S*. *halepense* seeds have a higher specific gravity than those of *R*. *crispus* and thus were recovered in higher numbers. Small seeds are expected to have a pattern similar of rate passage to that of the liquid fraction in a fermentational bag, whereas large seeds are expected to have the pattern similar to that of particulate matter. Thus, specific gravity has a greater influence on the rate of passage of small particles than that of the sieving effects of the mass of reticulo-rumen [38].

In all cattle groups, the highest seed recovery occurred two days after seed intake. Gökbulak (2006) also reported a similar peak time in Holstein heifers for seed recovery from three perennial species and two forbs species [5]. The length of time for 50% recovery of tropical pasture seeds after intake average over the ruminants (goat, sheep, and cattle) has been measured to be about 51–71 h [17] and in cattle it was 34–51 h [16]. The recovery rate for undamaged seed depends on the chewing style, which varies between ruminants, with sheep and goats causing more damage to seeds than cattle [17]. These results demonstrate that a wide range of seed excretion rates is likely to happen because of differences in animal diet and seed characteristics.

Several studies have demonstrated that the viability of excreted seeds declines with the length of time seeds spend in the digestive tract [17, 21, 39]. The seed coat and degree of seed hardness and dormancy are important factors in determining the viability of seeds passing through the digestive tract [16, 22]. Initial seed germination was lower in the three species with higher viability (4.3%, 0% and 3.2% in *C*. *campestris*, *P*. *aviculare* and *S*. *halepense* respectively) than in *R*. *crispus*, which had an initial germination as high as 87%. Impermeable seed coat of *C*. *campestris* prevents germination leading to physical dormancy in this species [40, 41], which may help it to survive the passage. These results suggest that seeds with higher dormancy could probably be more resistant to digestion. However, to test this hypothesis one needs to use seeds that vary in the degree of dormancy only but no other traits e.g. seeds from the same species but with different dormancy levels.

Feedlot male calves and lactating cows caused higher seed mortality than dry cows and growing heifers (Figs 4 and 5), whereas total recovered viable seed was highest in lactating cows followed by growing heifers (Fig 3). Ruminal pH varied from 6.2 for feedlot male calves to 7.4 for dry cows at 0700 h before the morning meal (Fig 7). A high feed intake as ad libitum, especially with a high level of concentrate, can cause fluctuation in ruminal pH, ammonia and volatile fatty acids (VFA) concentrates [20, 42–44]. Furthermore, a high proportion of wheat straw in dry cow diets can increase total chewing time, which in turn can lead to an increase in buffering conditions in the rumen. The level of NDF has a positive effect on increasing chewing activity and rumen buffering [43, 45]. These factors might have led to more seed loss observed in feedlot male calves and lactating cows over the third and fourth days after the seed intake. It seems that the timing of seed excretion is the preliminary factor affecting the seed survival whilst other factors such as pH and NDF became important once seeds persist in the digestive tract for a longer period. For example, lactating cows and growing heifers excreted a high amount of seeds within the first two days after feeding: this rapid excretion rate (i.e. small *t*_*R*50_) resulted in high total survival rate (Fig 3).

As lactating cows exhibited the highest recovered viable seeds (Fig 3), this group of Holstein cattle is more likely to infest cropland with manure rich in weed seeds than other cattle types. However, this hypothesis is based on the assumptions that weed seeds are distributed uniformly across all the feed types offered to cattle. Common practice in formulating cattle diets is based solely on the nutrient requirements of the herd and on production goals. However, if the manure of the cattle is to be used on farmlands, the risks associated with the spread of weed seeds from that manure also need to be considered.

Our findings suggest that weed seed fate can follow different trajectories depending on cattle types (of the same species) due to variation in animal physiology (e.g. gut pH) and diet (e.g. digestibility). Such variations need to be accounted for when formulating a diet and subsequently applying the manure on crop fields. Our study can be used as a guideline for evaluating the potential risk of the spread of weed seeds through the application of cattle manure, specifically in relation to Holstein cattle.

## Supporting Information

S1 Table Parameters and root measn squared error (RMSE) and adjusted coefficients estimated for the power model for viability of recovered seeds in foure groups of Holstein cattle. (PDF)
