# Supplementary material for: Variation in Weed Seed Fate Fed to Different Holstein Cattle Groups
Source: PLoS One. 2016 Apr 22;11(4):e0154057. doi: 10.1371/journal.pone.0154057 (PMC4841545; doi:10.1371/journal.pone.0154057)
Supplement: S1 Table — (PDF) [file pone.0154057.s001.pdf]

S1 Table. Parameters and root mean squared error (RMSE) and adjusted coefficients estimated for the power model for viability of recovered seeds in four groups of Holstein cattle.

		a	*b*	RMSE	R_{adj}^2
Groups	Weed species				
Lactating cow	*C. campestris*	-4.30 (0.90)	2.33 (0.20)	3.88	0.98
	P. aviculare	-2.69 (0.44)	2.59 (0.12)	3.97	0.98
	R. crispus	-5.31 (0.66)	2.36 (0.11)	2.68	0.98
	S. halepense	-5.84 (0.74)	2.00 (0.09)	4.14	0.98
Growing heifer	*C. campestris*	-2.61 (0.36)	2.49 (0.10)	3.09	0.98
	P. aviculare	-1.18 (0.19)	3.04 (0.12)	2.64	0.99
	R. crispus	-1.71 (0.71)	3.17 (0.38)	4.66	0.96
	S. halepense	-2.02 (0.25)	2.64 (0.09)	2.39	0.97
Feedlot male calf	*C. campestris*	-4.04 (0.81)	2.54 (0.19)	3.90	0.97
	P. aviculare	-2.67 (0.56)	2.84 (0.19)	3.07	0.98
	R. crispus	-4.26 (0.85)	2.58 (0.18)	3.78	0.96
	S. halepense	-5.45 (1.04)	2.25 (0.18)	4.04	0.98
Dry cow	*C. campestris*	-2.55 (0.39)	2.55 (0.12)	3.33	0.99
	P. aviculare	-0.68 (0.09)	3.49 (0.10)	2.02	0.99
	R. crispus	-1.26 (0.97)	3.48 (071)	7.88	0.98
	S. halepense	-0.97 (0.37)	3.25 (0.28)	6.02	0.99

Values in parentheses are standard errors.
